# The association between statistical shape modeling-defined hip morphology and features of early hip osteoarthritis in young adult football players: Data from the femoroacetabular impingement and hip osteoarthritis cohort (FORCe) study

**DOI:** 10.1016/j.ocarto.2022.100275

**Published:** 2022-05-20

**Authors:** M.M.A. van Buuren, J.J. Heerey, A. Smith, K.M. Crossley, J.L. Kemp, M.J. Scholes, P.R. Lawrenson, M.G. King, W.P. Gielis, H. Weinans, C. Lindner, R.B. Souza, J.A.N. Verhaar, R. Agricola

**Affiliations:** a Department of Orthopaedics and Sports Medicine, Erasmus MC, University Medical Center Rotterdam, Rotterdam, the Netherlands; b La Trobe Sport and Exercise Medicine Research Centre, School of Allied Health, Human Services and Sport, La Trobe University, Melbourne, Australia; c School of Physiotherapy and Exercise Science, Curtin University, Perth, Australia; d School of Health & Rehabilitation Sciences, The University of Queensland, Brisbane, Australia; e Department of Orthopaedics, University Medical Center Utrecht, Utrecht, the Netherlands; f Department of Biomechanical Engineering, Delft University of Technology, Delft, the Netherlands; g Division of Informatics, Imaging & Data Sciences, University of Manchester, Manchester, United Kingdom; h Department of Physical Therapy and Rehabilitation Science, University of California San Francisco, San Francisco, CA, USA

**Keywords:** Hip morphology, Femoroacetabular impingement, Statistical shape modeling, Hip osteoarthritis, Football, Magnetic resonance imaging

## Abstract

**Objective:**

To explore the relationship between radiographic hip shape and features of early hip osteoarthritis (OA) on magnetic resonance imaging (MRI) in young male and female football players without radiographic hip OA.

**Design:**

We used baseline data from a cohort of symptomatic and asymptomatic football players aged 18–50 years. Hip shape was assessed on anteroposterior radiographs with statistical shape modeling (SSM) for men and women separately. Cartilage defects and labral tears were graded using the Scoring Hip Osteoarthritis with MRI (SHOMRI) system. We used logistic regression with generalized estimating equations to estimate associations between each hip shape variant, called shape modes, and cartilage defects or labral tears.

**Results:**

We included 229 participants (446 hips, 77.4% male). For each sex, 15 shape modes were analyzed. In men, three shape modes were associated with cartilage defects: adjusted odds ratios (aOR) 0.75 (95%CI 0.58–0.97) per standard deviation (SD) for mode 1; 1.34 (95%CI 1.05–1.69) per SD for mode 12; and 0.61 (95%CI 0.48–0.78) per SD for mode 15; and one also with labral tears: aOR 1.30 (95%CI 1.01–1.69) per SD for mode 12. These modes generally represented variations in the femoral neck and subtypes of cam morphology, with and without pincer morphology. For women, there was no evidence for associations with the outcomes.

**Conclusions:**

Several hip shape variants were associated with cartilage defects on MRI in young male football players. Specifically, one subtype of cam morphology was associated with both cartilage defects and labral tears. Hip shape was not associated with early OA features in women.

## 1 Introduction

Osteoarthritis (OA) is a leading cause of physical impairment [1], and from all joints potentially affected by OA, the hip is associated with the highest level of disability [2,3]. Although the etiology of hip OA is not completely known, evidence points towards an important role for hip shape in the development of hip OA [4,5].

Prospective cohort studies consistently show an association between bony morphology (e.g., cam morphology and acetabular dysplasia) and the development of hip OA [[6], [7], [8], [9]]. Cam morphology is defined as an aspherical femoral head, which can lead to intra-articular damage when the cam morphology is repetitively forced into the acetabulum during motion. When painful, this is referred to as femoroacetabular impingement (FAI) syndrome [10]. Acetabular dysplasia is defined as undercoverage of the femoral head by the acetabulum. This can lead to higher peak forces in the cartilage, thereby increasing the risk of hip OA over time [6,11].

Shape variants other than cam morphology and acetabular dysplasia may also increase the risk of developing hip OA. Statistical shape modeling (SSM) is a technique that can quantify the total hip shape and identify all hip shape variants that exist in a given population. As SSM can capture all variations in hip shape, rather than measuring a single shape element, it is particularly suitable for hypothesis generation. A recent systematic review showed associations between certain SSM-defined radiographic hip shape variants and radiographic hip OA in older adults [4].

The relationship between hip shape and hip OA is typically studied in middle-aged and older populations, but some hip shape variants develop from birth (acetabular dysplasia) [12] or during adolescence (cam morphology) [[13], [14], [15]]. The pathways from certain hip shape variants to the development of hip OA may be reversible, or at least modifiable, with certain interventions. Therefore, it is important to recognize intra-articular changes, such as cartilage defects or labral tears, resulting from these variants at an early stage, as they are generally considered signs of early hip OA [16]. Little is known about the relationship between hip shape and signs of early hip OA in young adults. Cam morphology, as defined by the alpha angle, seems to be associated with cartilage and labral lesions [17,18]. However, the relationship between (SSM-defined) ‘general hip shape’ and these early features of hip OA in a young, athletic population is still unknown. In particular, athletes participating in high-impact sports might be prone to early hip OA in the presence of certain hip shape variations, including but not limited to cam morphology [[19], [20], [21]].

We therefore aimed to explore the relationship between SSM-defined radiographic hip shape and cartilage or labral lesions in young football players. Separate shape models were built for men and women, because of the known morphological differences between the sexes [22,23].

## 2 Methods

### 2.1 Study design

This cross-sectional study used baseline data from the femoroacetabular impingement and hip osteoarthritis cohort (FORCe) study. The FORCe study has been described in detail elsewhere [24]. In short, it is an ongoing prospective cohort study that aims to evaluate changes in hip joint structure in sub-elite soccer and Australian football players, with both symptomatic and asymptomatic hips. Participants were recruited in Melbourne and Brisbane (Australia) between August 2015 and October 2018. Ethics approvals have been obtained from La Trobe University Human Ethics Committee (HEC15-019 and HEC16-045) and from the University of Queensland Medical Research Ethics Committee (2015000916 and 2016001694), and all participants have given their written informed consent. This study was conducted in compliance with the Declaration of Helsinki.

### 2.2 Participants

A full list of eligibility criteria for participants is given in Supplementary Table S1. Participants were sub-elite level soccer or Australian football players aged between 18 and 50 years. Hips were classified by symptomatic status as previously described [20]. In short, symptomatic participants had one or two symptomatic hips, defined by self-reported hip and/or groin pain for more than 6 months and a positive flexion-adduction-internal rotation (FADIR) test. If the contralateral hip was asymptomatic or had a negative FADIR test, it was included as “other hip”. In the asymptomatic participants, both hips were asymptomatic and had a negative FADIR test. This resulted in three possible symptomatic states for the hips: symptomatic, asymptomatic, and other. For building the shape models, all hips were merged into one group, but we adjusted for symptomatic status in the analyses.

### 2.3 Radiography

All participants underwent a supine anteroposterior (AP) pelvic radiograph with their legs in 15° internal rotation, using a standardized protocol [20]. Each radiograph was evaluated for individual radiographic features of hip OA using the Osteoarthritis Research Society International (OARSI) atlas by an orthopedic surgeon (RA) who was blinded to the participants’ clinical presentation [25]. A summary Kellgren-Lawrence (KL) grade [26] was derived from the individual features. Intraobserver reliability scores were calculated by re-reading 20 randomly selected radiographs 6 months after the initial reading. The intraobserver agreement for KL grading was strong, with a kappa of 0.87 (95% CI 0.71–1.00) [27]. Participants with doubtful hip OA or worse (i.e., Kellgren-Lawrence (KL) grade >0) were excluded from this study, because radiographic OA signs could potentially alter hip shape.

### 2.4 Statistical shape modeling

We used SSM to quantify the apparent radiographic hip shape of participants using their AP pelvic radiographs. SSM is a method to quantify the whole shape of an object, in this case the hip joint, rather than measuring individual features such as angles or indices. This was done by annotating the bony contours of the hip joint on a set of radiographs using a standardized set of radiographic landmark points. The shapes were then aligned with Generalized Procrustes analysis to remove variation in position, scale and orientation, and analyzed with principal components analysis. This identified the mean shape and the main shape variations, called shape modes, within the study population. The first shape mode contributes most to the total shape variation, with subsequent shape modes each contributing less than the previous mode. However, a lower percentage of variance explained does not mean that the variation is less prevalent in the population.

For this study, we used an automatic segmentation system: BoneFinder® (Manchester, United Kingdom) [[28], [29], [30]], to annotate each radiograph with 75 landmark points. This automatic segmentation system was trained with a set of manually annotated radiographs from the Cohort Hip and Cohort Knee (CHECK) study [31]. After running the automatic annotation, we manually added 7 points around the femoral head-neck junction, and visually verified and (if needed) fine-tuned the other points, creating an SSM with 82 points. Because there are evident hip shape variations between sexes [22,23], we created two different SSMs for men and women, and analyzed these separately. We standardized the shape mode variables produced by the SSM to have a mean of 0 and a standard deviation (SD) of 1. This was done so that the statistical unit of change was always one SD, which provides better interpretation of results. We limited the number of investigated hip shape modes by only including hip shape modes that explained at least 1.00% of the total shape variation within the studied population. This percentage was arbitrarily picked to reduce multiple testing and to only investigate (clinically) relevant shape modes.

### 2.5 Magnetic resonance imaging and outcomes

Participants underwent unenhanced 3.0 ​T magnetic resonance imaging (MRI) (Philips Ingenia, The Netherlands) of both hips independently, using a standardized protocol [20]. MRI scans were evaluated by an experienced musculoskeletal radiologist, blinded to clinical and radiological findings. Cartilage defects and labral tears were scored as part of the scoring hip osteoarthritis with MRI (SHOMRI) system [32]. Cartilage defects were graded 0 (no loss), 1 (partial-thickness loss), or 2 (full-thickness loss), for ten subregions: four acetabular and six femoral. Labral tears were graded 0 (normal), 1 (abnormal signal or fraying), 2 (simple tear), 3 (labro-cartilage separation), 4 (complex tear), or 5 (maceration), for four subregions. For the current cross-sectional analysis, we dichotomized these outcomes: a cartilage defect was considered present if any cartilage loss (SHOMRI grade ≥1) was evident in at least one acetabular or femoral subregion, and a labral tear was considered present if a SHOMRI grade ≥2 lesion was found in any subregion. We considered cartilage defects and labral tears to be the most representative features for early hip OA [33], so excluded the other SHOMRI features in this study. Intra-observer reliability scores were calculated by re-reading 20 randomly selected hip MRIs two weeks after the initial scoring. Intraobserver agreement for SHOMRI outcomes was moderate, with a kappa of 0.66 (95% CI 0.54–0.78) for presence of any cartilage defects; and a kappa of 0.77 (95% CI 0.62–0.92) for labral tears [20].

### 2.6 Statistical analysis

For the primary aim, we analyzed the associations between male and female hip shape modes and features of early hip OA at per hip level, using logistic regression models with generalized estimating equations to adjust for within-person correlation between right and left hips. The dependent variables were the presence or absence of cartilage defects or labral tears, and the independent variables were the standardized shape mode values from the SSMs. We adjusted for age, body mass index (BMI), and symptomatic status of the hips. Due to within-person correlation, we kept the distinction between asymptomatic hips and “other hips” (the asymptomatic contralateral hips in symptomatic participants). We did not perform an a priori power analysis because this was a hypothesis-free exploratory study. Estimates of associations between hip shape modes and cartilage defects or labral tears are presented as odds ratios (OR) per 1 SD increase in standardized shape mode value, with 95% confidence intervals (CI) and p-values. An OR > 1.00 means that an increase in shape mode value is associated with the outcome, while an OR < 1.00 means that a decrease in shape mode value is associated with the outcome. We additionally reported the Bonferroni alpha to adjust the significance level to 0.05 divided by the number of tested shape modes. Due to the exploratory nature of this study, and to lower the chance of type II errors, we described all associations with p ​< ​0.05. Analyses were performed using IBM SPSS Statistics, Version 25.0 (IBM Corp., Armonk, New York, USA).

## 3 Results

### 3.1 Participants

The FORCe study included a total of 239 participants: 184 with at least one symptomatic hip and 55 with two asymptomatic hips. Complete baseline data, including an AP pelvic radiograph suitable for SSM and hip MRI scans, were available for 236 participants (465 hips). After exclusion of hips with KL grade >0 (19 hips), 229 participants (446 hips, 345 male and 101 female) remained for analysis. Characteristics of the included participants and hips are shown in Table 1. Cartilage defects were present in 51.9% of male and 33.7% of female hips. Labral tears were present in 70.4% of male and in 68.3% of female hips.

**Table 1:** Characteristics of included participants and hips. Values are median [Q1 – Q3] or number (percentage). BMI ​= ​body mass index.

Participant-level characteristics (n ​= ​229)	Men (n ​= ​178)	Women (n ​= ​51)
Age, years	26 [23–31]	25 [22–29]
Height, m	1.81 [1.77–1.86]	1.66 [1.64–1.72]
Mass, kg	80.1 [75.2–88.7]	63.1 [57.3–71.8]
BMI, kg/m^2^	24.5 [23.0–26.5]	22.7 [21.1–24.3]
Sport type		
Soccer	90 (50.6%)	25 (49.0%)
Australian football	88 (49.4%)	26 (51.0%)
Hip-level characteristics (n ​= ​446)	Men (n ​= ​345)	Women (n ​= ​101)
Symptomatic status		
Symptomatic hip (of symptomatic participant)	217 (62.9%)	59 (58.4%)
Other hip (of symptomatic participant)	54 (15.7%)	15 (14.9%)
Asymptomatic hip	74 (21.4%)	27 (26.7%)
Cartilage defect present	179 (51.9%)	34 (33.7%)
Labral tear present	243 (70.4%)	69 (68.3%)

### 3.2 Statistical shape model

For both the male and the female SSM, the first 15 hip shape modes each explained at least 1.0% of the total shape variation. In men, the first 15 shape modes together explained 83.2% of the total shape variation, while in women the first 15 shape modes explained 86.5% of total variation. The list of extracted shape modes and the explained variance per mode can be found in Table 2. Graphical representations of each hip shape mode can be found in Supplementary Figure S1 (men) and Supplementary Figure S2 (women), and the authors’ descriptions of each shape mode can be found in Supplementary Tables S2 and S3. Note that descriptions can be subjective.

**Table 2:** Extracted hip shape modes and percentage of variance explained per mode.

Shape Mode	Male shape model	Female shape model
	Percentage of variance explained (cumulative)	Percentage of variance explained (cumulative)
Mode 1	25.5% (25.5%)	25.2% (25.2%)
Mode 2	13.3% (38.7%)	15.2% (40.5%)
Mode 3	10.6% (49.4%)	9.8% (50.2%)
Mode 4	7.2% (56.6%)	7.9% (58.2%)
Mode 5	5.4% (62.0%)	5.9% (64.1%)
Mode 6	4.4% (66.4%)	5.0% (69.1%)
Mode 7	3.4% (69.8%)	3.3% (72.4%)
Mode 8	2.5% (72.3%)	2.9% (75.3%)
Mode 9	2.3% (74.7%)	2.5% (77.7%)
Mode 10	2.0% (76.7%)	2.2% (79.9%)
Mode 11	1.8% (78.4%)	1.7% (81.6%)
Mode 12	1.4% (79.9%)	1.4% (83.0%)
Mode 13	1.2% (81.1%)	1.3% (84.3%)
Mode 14	1.1% (82.2%)	1.2% (85.5%)
Mode 15	1.0% (83.2%)	1.0% (86.5%)

### 3.3 Associations between male shape variations and cartilage defects

**Fig. 1:**
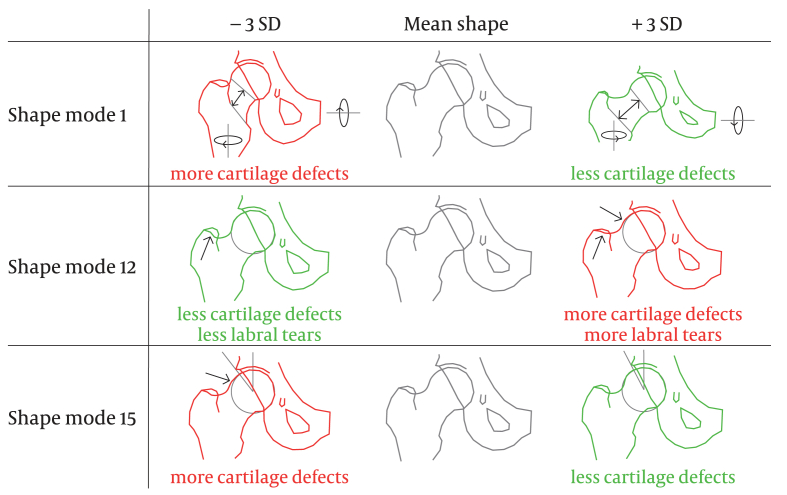
Male shape modes that showed a statistically significant association with cartilage defects, labral tears, or both. The shape mode in grey is the mean shape in men. Shape modes in red are associated with higher prevalence of the outcome(s), while shape. modes in green are associated with lower prevalence of the outcome(s). SD ​= ​standard deviation.

Within the male SSM, there was evidence for an association with the presence of cartilage defects for three out of the 15 analyzed shape modes (Fig. 1). All estimates of associations between male hip shape modes and cartilage defects are presented in Table 3. Shape mode 1 had an adjusted OR of 0.75 per SD increase (95% CI 0.58–0.97) for cartilage defects. Negative mode 1 values (associated with the outcome) appear to represent a short femoral neck with prominent trochanters, low offset, valgus orientation, and posterior pelvic tilt. Shape mode 12 had an adjusted OR of 1.34 per SD increase (95% CI 1.05–1.69) for cartilage defects. Positive mode 12 values seem to represent a cam morphology and a less protruding greater trochanter. Shape mode 15 had an adjusted OR of 0.61 per SD increase (95% CI 0.48–0.78) for cartilage defects. Negative mode 15 values seem to represent a cam morphology with overcoverage of the acetabulum (pincer morphology). Only the association between shape mode 15 and the presence of cartilage defects would still be statistically significant if Bonferroni correction was applied (*P* ​= ​0.0001).

**Table 3:** Associations between male hip shape modes and the presence of a cartilage defect or a grade 2 or higher labral tear on MRI.

Male shape Model	Outcome: Cartilage defects				Outcome: Labral tears			
Shape Mode	OR (95% CI)	*P*-Value	aOR (95% CI)	*P*-Value	OR (95% CI)	*P*-Value	aOR (95% CI)	*P*-Value
Mode 1	0.73 (0.57–0.95)	0.018^a^	0.75 (0.58–0.97)	0.026^a^	0.86 (0.66–1.11)	0.245	0.84 (0.65–1.10)	0.215
Mode 2	0.85 (0.68–1.06)	0.150	0.87 (0.69–1.10)	0.238	0.96 (0.75–1.22)	0.742	0.98 (0.76–1.27)	0.890
Mode 3	1.11 (0.88–1.41)	0.385	1.13 (0.89–1.44)	0.319	1.24 (0.97–1.59)	0.088	1.25 (0.97–1.61)	0.087
Mode 4	1.04 (0.83–1.30)	0.743	1.01 (0.80–1.27)	0.965	0.97 (0.76–1.22)	0.784	0.96 (0.75–1.22)	0.734
Mode 5	0.88 (0.69–1.12)	0.291	1.01 (0.58–1.77)	0.962	0.93 (0.74–1.18)	0.574	0.91 (0.73–1.15)	0.441
Mode 6	1.10 (0.88–1.36)	0.401	1.08 (0.87–1.35)	0.480	1.14 (0.91–1.43)	0.251	1.18 (0.94–1.49)	0.160
Mode 7	1.12 (0.91–1.37)	0.301	1.12 (0.91–1.38)	0.275	1.04 (0.81–1.33)	0.770	1.05 (0.82–1.34)	0.701
Mode 8	1.05 (0.85–1.31)	0.655	1.03 (0.82–1.28)	0.826	0.95 (0.73–1.24)	0.722	0.93 (0.72–1.22)	0.612
Mode 9	1.10 (0.86–1.39)	0.458	1.06 (0.83–1.35)	0.658	0.98 (0.77–1.26)	0.879	0.96 (0.74–1.25)	0.775
Mode 10	0.87 (0.71–1.08)	0.205	0.85 (0.69–1.05)	0.137	0.88 (0.69–1.11)	0.279	0.87 (0.68–1.11)	0.272
Mode 11	0.92 (0.73–1.16)	0.471	0.91 (0.72–1.15)	0.450	0.98 (0.77–1.24)	0.862	0.98 (0.77–1.24)	0.850
Mode 12	1.34 (1.06–1.70)	0.013^a^	1.34 (1.05–1.69)	0.017^a^	1.28 (1.00–1.65)	0.052	1.30 (1.01–1.69)	0.046^a^
Mode 13	0.87 (0.69–1.09)	0.216	0.91 (0.72–1.15)	0.415	0.86 (0.65–1.15)	0.312	0.85 (0.64–1.14)	0.286
Mode 14	1.00 (0.82–1.23)	0.973	1.00 (0.81–1.23)	0.983	0.95 (0.75–1.20)	0.659	0.95 (0.75–1.21)	0.688
Mode 15	0.62 (0.49–0.78)	0.0001^b^	0.61 (0.48–0.78)	0.0001^b^	0.85 (0.68–1.06)	0.146	0.84 (0.68–1.05)	0.133

All odds ratios (OR) are per 1 SD increase in shape mode value.

Adjusted OR's (aOR) are adjusted for age, body mass index and symptomatic status.

Bonferroni alpha ​= ​0.05/15 ​= ​0.0033.

CI ​= ​confidence interval.

a Statistically significant at the 0.05 level.

b Statistically significant at the 0.0033 level if Bonferroni correction would be applied.

### 3.4 Associations between male shape variations and labral tears

Estimates of associations between male hip shape modes and labral tears are presented in Table 3. For one of the 15 male hip shape modes (shape mode 12) there was evidence of an association with labral tears (adjusted OR 1.30, 95% CI 1.01–1.69). This association would not be statistically significant when applying the Bonferroni correction (*P* ​= ​0.046). This shape variation was also associated with cartilage defects, as described above.

None of the 15 hip shape modes within the female SSM was statistically significantly associated with cartilage defects or labral tears after adjustment for age, BMI and symptomatic status. Estimates of associations between female hip shape modes and cartilage defects and labral tears are presented in Table 4.

**Table 4:** Associations between female hip shape modes and the presence of a cartilage defect or a grade 2 or higher labral tear on MRI.

Female shape model	Outcome: Cartilage defects				Outcome: Labral tears			
Shape Mode	OR (95% CI)	*P*-Value	aOR (95% CI)	*P*-Value	OR (95% CI)	*P*-Value	aOR (95% CI)	*P*-Value
Mode 1	1.38 (0.81–2.34)	0.241	1.63 (0.92–2.88)	0.096	0.76 (0.47–1.24)	0.278	0.77 (0.45–1.32)	0.350
Mode 2	0.99 (0.67–1.46)	0.943	0.89 (0.55–1.42)	0.620	1.11 (0.67–1.85)	0.688	1.15 (0.65–2.04)	0.636
Mode 3	0.86 (0.57–1.32)	0.493	0.81 (0.51–1.30)	0.384	1.18 (0.74–1.88)	0.500	1.11 (0.67–1.84)	0.672
Mode 4	0.85 (0.51–1.40)	0.516	0.88 (0.52–1.49)	0.643	0.68 (0.44–1.05)	0.084	0.67 (0.42–1.08)	0.100
Mode 5	1.07 (0.68–1.70)	0.763	1.02 (0.65–1.62)	0.920	1.10 (0.71–1.70)	0.674	1.10 (0.66–1.83)	0.713
Mode 6	1.05 (0.67–1.64)	0.821	1.00 (0.63–1.60)	0.985	1.02 (0.65–1.58)	0.941	0.97 (0.59–1.59)	0.892
Mode 7	1.02 (0.65–1.60)	0.939	1.03 (0.64–1.66)	0.897	0.98 (0.60–1.59)	0.921	0.99 (0.58–1.69)	0.970
Mode 8	1.21 (0.80–1.85)	0.370	1.24 (0.81–1.89)	0.315	0.87 (0.56–1.34)	0.521	0.85 (0.53–1.36)	0.491
Mode 9	1.28 (0.86–1.92)	0.225	1.26 (0.84–1.90)	0.266	1.31 (0.77–2.24)	0.316	1.26 (0.68–2.34)	0.471
Mode 10	0.96 (0.60–1.52)	0.858	0.98 (0.59–1.60)	0.925	0.90 (0.60–1.35)	0.599	0.80 (0.51–1.25)	0.329
Mode 11	0.80 (0.55–1.15)	0.223	0.81 (0.53–1.23)	0.314	1.15 (0.73–1.81)	0.535	1.17 (0.75–1.81)	0.487
Mode 12	1.12 (0.73–1.73)	0.598	1.08 (0.68–1.73)	0.734	1.02 (0.67–1.56)	0.925	1.04 (0.68–1.59)	0.859
Mode 13	1.16 (0.71–1.91)	0.544	1.21 (0.69–2.10)	0.505	0.90 (0.57–1.42)	0.656	0.93 (0.57–1.49)	0.752
Mode 14	1.07 (0.68–1.68)	0.758	1.08 (0.66–1.75)	0.766	0.83 (0.53–1.32)	0.436	0.84 (0.52–1.35)	0.474
Mode 15	0.64 (0.39–1.03)	0.064	0.65 (0.40–1.06)	0.086	0.58 (0.34–0.98)	0.043^a^	0.62 (0.37–1.05)	0.075

All odds ratios (OR) are per 1 SD increase in shape mode value.

Adjusted OR's (aOR) are adjusted for age, body mass index and symptomatic status.

CI ​= ​confidence interval.

a Statistically significant at the 0.05 level.

## 4 Discussion

In male football players without radiographic hip OA, three SSM-defined radiographic hip shape variations were associated with cartilage defects, and one of those was also associated with labral tears. Two shape modes that showed significant associations with the outcomes in men seemed to represent types of cam morphology, while multiple other shape modes also seemingly representing cam morphology showed no significant associations, suggesting that different subtypes of cam morphology might exist.

The shape variation that explained the most variance (mode 1) seemed to represent prominent trochanters and a short femoral neck (possibly also explained by position-related external rotation of the leg, rather than anatomical variation), a high neck-shaft angle (valgus hip), and posterior pelvic tilt. The adjusted OR for this association was 0.75 (95% CI 0.58–0.97) per SD increase in shape mode value, meaning that for every one SD decrease in mode 1 value, the odds for the presence of a cartilage defect increases by 1.33 (1/0.75). Participants that scored −3 SD for this shape mode, would then have an OR of 2.37 (1.33^3) compared to participants with a mean hip shape. The other two shape variations that were associated with cartilage defects both seemed to represent cam morphology (mode 12 and mode 15), while one showed pincer morphology too (mode 15). Mode 15 had an adjusted OR of 0.61 (0.48–0.78) per SD, meaning that a participant scoring −3 SD on this shape mode would have an OR of 4.41 ((1/0.61)^3). The other shape mode featuring cam morphology (mode 12) was also associated with labral tears in men. There was no evidence of associations between any female shape mode and cartilage defects or labral tears.

Besides the two cam-type shape variations that were associated with the outcomes, the male shape model showed multiple other shape variations that also resembled cam morphology (Supplementary Figure S1). These other shape modes appear to have high alpha angles too, although they were not associated with cartilage defects. This may suggest that different subtypes of cam morphology may pose different risks for cartilage defects, and eventually for hip OA. Another possible explanation is that the risk comes with a combination of shape features, and not just cam morphology. For example, shape mode 12 (associated with cartilage defects and labral tears) features a pistol-grip cam morphology, but also a wider femoral neck and a flatter greater trochanter. Shape mode 15 (associated with cartilage defects only) represents a pistol-grip cam morphology, but also features a wider femoral neck and some overcoverage of the acetabulum (pincer morphology). As cam and pincer morphology were both present, this shape mode could represent mixed morphology. We recommend that future studies should not only focus on cam morphology, as measured by the alpha angle, but could look either at subtypes of cam morphology or at the entire hip joint shape, including the acetabulum or even the pelvic anatomy and orientation.

Shape modes in women did not feature cam morphology or apparent high alpha angles, suggesting a distinctly different bony morphology than that from men (Supplementary Figure S2). The mean hip shape was also different between the sexes, with the mean male hip shape showing a more prominent superior femoral head-neck junction, while the mean female shape features a more spherical femoral head. This reinforces the need for separate shape models for the sexes. Previous studies have also shown a low prevalence of cam morphology in females [34]. In our study, the prevalence of cartilage defects was also lower in women than in men, which could potentially be explained by the lower prevalence of cam morphology in women.

Our findings are consistent with other literature on the relationship between cam morphology (alpha angle ≥60) and various measures of early hip OA (i.e., delayed gadolinium-enhanced MRI [[35], [36], [37], [38]] and T1 rho MRI [[39], [40], [41]]). However, our study adds to the current literature, because we distinguished different subtypes of cam morphology and included other shape variants through SSM. This is also the main strength of our study: we did not predefine any shape variations, and therefore investigated hypothesis-free associations between general radiographic hip shape and cartilage defects and labral tears.

One limitation of our study is that it included relatively few women compared to men. The female SSM was generated from 101 hips, so it is not clear how representative the shape modes are of female high-impact athletes in general. Moreover, with the lower numbers of cartilage defects (34) and labral tears (69), this study may have been underpowered for detecting associations in women. The minimal detectable OR for the outcome cartilage defects in women (for a continuous predictor with the current sample size and 80% power) was 1.89 (or 0.53 for lower odds) per SD change in shape mode value [42]. This means that anything below this value was unlikely to have been detected in our study. Another limitation may be that all SHOMRI scoring was done by one musculoskeletal radiologist, and we did not assess inter-observer agreement. Therefore, the presence and severity of cartilage defects and/or labral tears may have been over- or underestimated. A drawback of exploratory studies like these is the increased chance of a type I error due to multiple testing. However, strictly applying corrections like the Bonferroni correction would increase the chance of type II errors. Therefore, we decided to provide the Bonferroni alpha without using it strictly. A relative limitation is the quantification of hip shape on AP pelvic radiographs only: while AP pelvic radiographs are inexpensive and widely used, the complete 3-dimensional shape of the hip is not captured on a 2-dimensional projection. On top of that, the found shape modes may not only represent anatomical shape variations, but also apparent variations due to subject positioning and radiographic projection effects. However, given the widespread use of conventional radiography for hip-related disease, we feel that the results are still relevant. Lastly, because participants were recruited through local ads and information sessions, the sample population may not be entirely representative of all young adult soccer and Australian football players.

In conclusion, several hip shape variations regarding orientation of the femoral neck or subtypes of cam morphology (with or without pincer) were associated with cartilage defects on MRI in male football players without radiographic hip OA. One of the subtypes of cam morphology was associated with both cartilage defects and labral tears. No associations between hip shape and early hip OA were found in female football players.

## Contributions

Conception and design: MvB, JH, AS, KC, JLK, MS, PL, MK, RA, Analysis and interpretation of the data: MvB, JH, AS, KC, JLK, MS, PL, MK, WPG, HW, CL, RS, JV, RA, Drafting of the article: MvB, RA, Critical revision of the article for important intellectual content: MvB, JH, AS, KC, JLK, MS, PL, MK, WPG, HW, CL, RS, JV, RA, Final approval of the article: MvB, JH, AS, KC, JLK, MS, PL, MK, WPG, HW, CL, RS, JV, RA, Provision of study materials or patients: JH, KC, JLK, MS, PL, MK, Collection and assembly of data: MvB, JH, JLK, MS, PL, MK, Statistical expertise: MvB, AS, CL, RA, Obtaining of funding: AS, KC, RA. The corresponding author (MvB) takes responsibility for the integrity of the work as a whole, from inception to finished article.

### Role of the funding source

The study was supported by a 10.13039/501100000925National Health and Medical Research Council of Australia (NHMRC) project grant (GNT1088683). The funding body did not have a role in study design, collection, analysis and interpretation of data, writing of the manuscript or decision to submit the manuscript for publication.

## Financial support

MvB is supported by a research grant from the Dutch Arthritis Society (18-2-203). JH, MK, MS are supported by the La Trobe University Postgraduate Research Scholarship. KC is supported in part by funding from a NHMRC project grant (GNT1088683). CL is supported by a research grant from the Medical Research Council, UK (MR/S00405X/1). RA is supported by an Erasmus MC research fellowship, a research grant from the Dutch Arthritis Society (21-1-205), a research grant from the Dutch Research Council (09150161910071), and the Anna Foundation.

## Declaration of competing interest

The authors declare they have no conflicts of interest.
