# Supplementary material for: The association between statistical shape modeling-defined hip morphology and features of early hip osteoarthritis in young adult football players: Data from the femoroacetabular impingement and hip osteoarthritis cohort (FORCe) study
Source: Osteoarthr Cartil Open. 2022 May 20;4(3):100275. doi: 10.1016/j.ocarto.2022.100275 (PMC9718108; doi:10.1016/j.ocarto.2022.100275)
Supplement: Multimedia component 1 [file mmc1.pdf]

Table S1
Eligibility criteria for the femoroacetabular impingement and hip osteoarthritis cohort study.

	Symptomatic group	Asymptomatic group
**Inclusion criteria**	• Age: 18 to 50 years<br>• Playing in a sub-elite football competition<br>• Undertaking at least 2 sessions (games or training) of soccer/Australian football per week<br>• Self-reported hip (anterior/lateral/posterior) and/or groin pain, for more than six months, with a gradual onset, scoring >3 and <8 on an 11-point NPRS with football or football-specific movements, with or without mechanical symptoms (clicking, giving way, locking or catching)<br>• Positive FADIR test in at least one hip	• Age: 18 to 50 years<br>• Playing in a sub-elite football competition<br>• Undertaking at least 2 sessions (games or training) of soccer/Australian Football per week<br>• Negative FADIR test in both hips
**Exclusion criteria**	• Self-reported history of significant hip or groin condition, specifically: bursitis, congenital dislocation of the hip, fractures, osteochondritis dissecans, Legg-Calvé-Perthes disease, septic or rheumatoid arthritis, slipped capital femoral epiphysis or subluxations/dislocations<br>• Previous hip, groin or pelvic surgery<br>• KL grade 2 or greater on AP pelvic radiograph<br>• Any lumbar spine or lower limb injury/complaint in the previous 3 months (i.e. hamstring muscle injury or sprained ankle) that resulted in the inability to weight-bear fully or undertake testing procedures<br>• Contra-indications to radiographs (e.g., pregnancy) or MRI (e.g., claustrophobia)<br>• Received intra-articular hip injection (of any type) in the previous 3 months<br>• Unable to understand spoken and written English	• Self-reported history of hip and/or groin pain, or significant hip or groin condition (see exclusion criteria for symptomatic participants)<br>• Past history of lower limb surgery (e.g. anterior cruciate ligament reconstruction)<br>• KL grade 2 or greater on AP pelvic radiograph<br>• Any lumbar spine or lower limb injury/complaint in the previous 3 months (e.g.<br>• hamstring muscle injury or sprained ankle) that resulted in the inability to weight-bear fully or undertake testing procedures<br>• Contra-indications to radiographs (i.e. pregnancy) or MRI (i.e. claustrophobia)<br>• Unable to understand spoken and written English

NPRS = Numeric Pain Rating Scale; FADIR = Flexion-Adduction-Internal Rotation; KL = Kellgren-Lawrence; AP = anteroposterior, MRI = magnetic resonance imaging;

Table S2
Authors' descriptions of shape modes from the male shape model.

Male shape model	Description of shape mode
Mode 1	Rotation of femur, neck-shaft angle, pelvic tilt, apparent acetabular coverage, apparent femoral neck length
Mode 2	Sphericity of femoral head (cam morphology), neck-shaft angle, prominence of greater trochanter, acetabular coverage
Mode 3	Size of femoral head, femoral neck length, prominence of greater trochanter, acetabular coverage, pelvic tilt
Mode 4	Sphericity of femoral head (cam morphology), neck-shaft angle, apparent size of lesser trochanter, acetabular depth
Mode 5	Sphericity of femoral head (cam morphology), acetabular coverage, width of greater trochanter
Mode 6	Sphericity of femoral head (cam morphology), shape of pubic bones
Mode 7	Femoral neck length, prominence of greater trochanter, pelvic tilt
Mode 8	Femoral neck width, greater trochanter width
Mode 9	Sphericity of femoral head (cam morphology), prominence of greater trochanter, shape of acetabular roof
Mode 10	Sphericity of femoral head (cam morphology), neck-shaft angle, prominence of greater and lesser trochanters
Mode 11	Sphericity of femoral head (cam morphology), size of lesser trochanter, acetabular coverage, shape of posterior acetabular wall
Mode 12	Sphericity of femoral head (cam morphology), prominence of greater trochanter
Mode 13	Size of femoral head, shape of posterior acetabular wall
Mode 14	Shape of teardrop, prominence of greater trochanter
Mode 15	Sphericity of femoral head (cam morphology), acetabular coverage

Table S3
Authors' descriptions of shape modes from the female shape model.

Female shape model	Description of shape mode
Mode 1	Neck-shaft angle, sphericity of femoral head, size of greater and lesser trochanters, pelvic tilt
Mode 2	Femoral neck length and width, size of greater and lesser trochanters, acetabular coverage and depth, pelvic tilt
Mode 3	Femoral neck length and width, neck-shaft angle, acetabular depth, pelvic tilt
Mode 4	Neck-shaft angle, acetabular coverage and depth, pelvic tilt
Mode 5	Size of lesser trochanter, acetabular coverage and depth, pelvic tilt
Mode 6	Sphericity of femoral head, acetabular coverage, teardrop shape
Mode 7	Sphericity of femoral head, femoral neck length and width
Mode 8	Femoral neck length and width, size of lesser trochanter, acetabular coverage, pelvic tilt
Mode 9	Prominence of greater trochanter, size of lesser trochanter
Mode 10	Sphericity of femoral head, femoral neck width, neck-shaft angle, acetabular coverage
Mode 11	Shape of acetabular roof, shape of lesser trochanter
Mode 12	Size of femoral head, prominence of greater and lesser trochanters
Mode 13	Femoral head shape, femoral neck width, prominence of greater trochanter
Mode 14	Sphericity and size of femoral head, femoral neck length, prominence of greater and lesser trochanters, acetabular depth
Mode 15	Lesser trochanter size, teardrop shape, shape of posterior acetabular wall

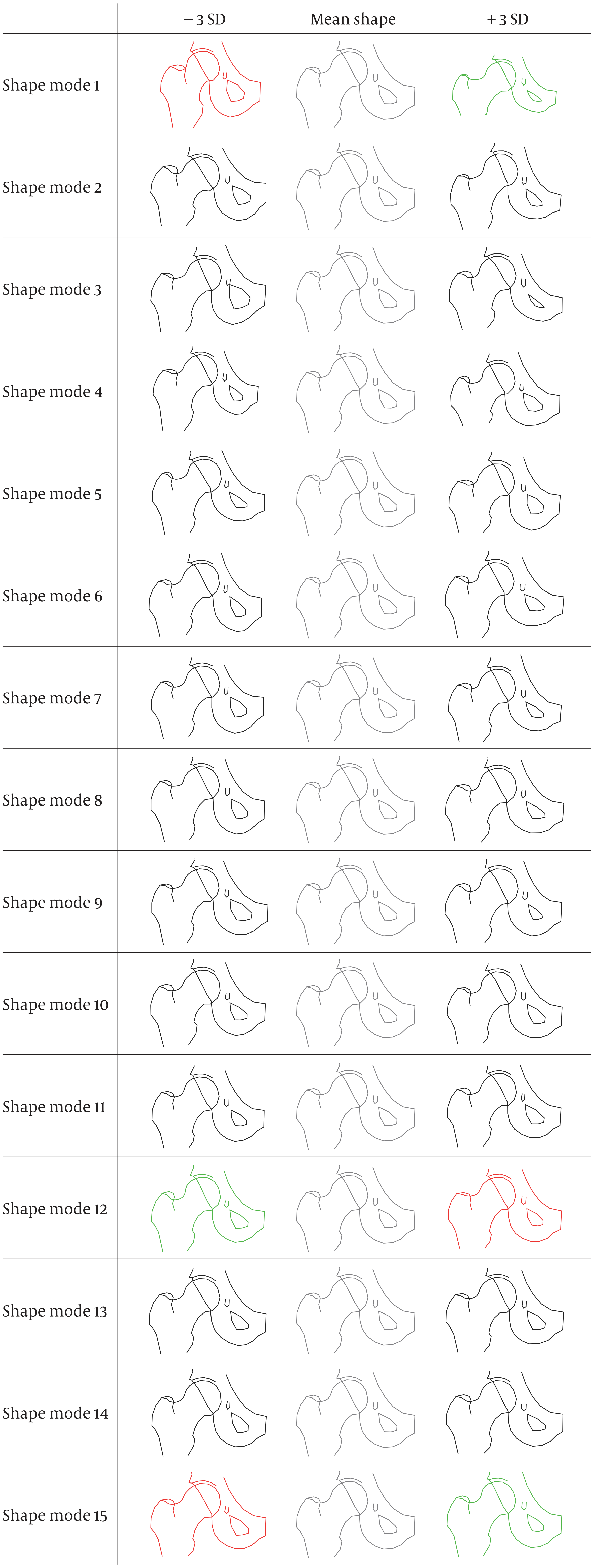

Fig. S1. All male shape modes that were included in the analysis. The shape mode in grey is the mean shape in men. Shape modes in red are associated with higher prevalence of the outcome(s), while shape modes in green are associated with lower prevalence of the outcome(s). Shape modes in black were not significantly associated with the outcomes.
SD = standard deviation.

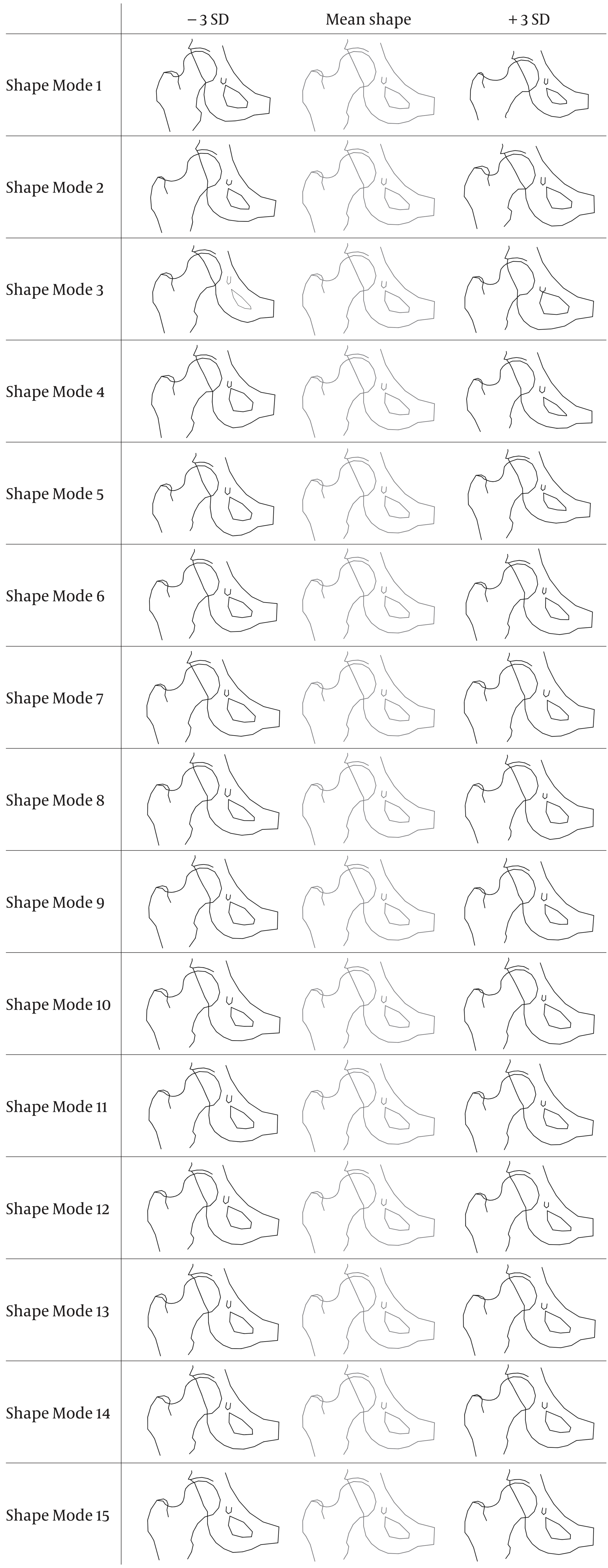

Fig. S2. All female shape modes that were included in the analysis. The shape mode in grey is the mean shape in women. Shape modes in black were not significantly associated with the outcomes after adjusting for covariates.
SD = standard deviation.
